# Performance of Metagenomic Next-Generation Sequencing for the Diagnosis of Cryptococcal Meningitis in HIV-Negative Patients

**DOI:** 10.3389/fcimb.2022.831959

**Published:** 2022-04-21

**Authors:** Zhouqing Gan, Jia Liu, Yijie Wang, Lu Yang, Zheng Lou, Han Xia, Min Li, Zhuolin Chen, Ying Jiang, Fuhua Peng

**Affiliations:** ^1^ Department of Neurology, The Third Affiliated Hospital of Sun Yat-sen University, Guangzhou, China; ^2^ Department of Scientific Affairs, Hugobiotech Co., Ltd., Beijing, China

**Keywords:** metagenomic next-generation sequencing, cerebrospinal fluids, cryptococcal meningitis, diagnosis, *Cryptococcus gattii*

## Abstract

**Objectives:**

Metagenomic next-generation sequencing (mNGS) has been applied more and more widely for the diagnosis of infectious diseases, but its performance in the diagnosis of cryptococcal meningitis (CM) remains unclear.

**Methods:**

Cerebrospinal fluid (CSF) samples from 197 HIV-negative patients with suspected central nervous system infections were tested simultaneously by mNGS and routine methods [India ink staining, fungal culture, or cryptococcal antigen (CrAg) tests]. The performance of mNGS was evaluated.

**Results:**

Of the 197 enrolled cases, 46 (23.4%) cases were finally diagnosed with CM, including 43 (93.5%) *Cryptococcus neoforman*s infections and 3 (6.5%) *Cryptococcus gattii* infections. The sensitivity, specificity, positive predictive value, negative predictive value, and concordance rate of mNGS were 93.5% [95% confidence interval (CI) at 86.4%~100.0%], 96.0% (95% CI at 92.9%~99.1%), 87.8%, 98.0%, and 95.4%, respectively. Comparing to the conventional diagnostic methods, the sensitivity and concordance rate of mNGS were slightly lower than those of CrAg tests (97.4%) but higher than those of India ink (63.0%) and culture (76.7%). Besides, mNGS had a sensitivity of 100.0% against culture. It should be noted that mNGS could identify *Cryptococcus* at species level; *C. gattii* of the 3 cases was only distinguished by mNGS.

**Conclusions:**

CSF mNGS can be considered as a supplementary test to diagnose CM and directly distinguish *C. gattii* from *C. neoforman*s in clinical specimens.

## Introduction

Culture of cerebrospinal fluid (CSF) is the “gold standard” for the diagnosis of cryptococcal meningitis (CM) (Perfect et al., 2010), a serious opportunistic fungal infection caused by *Cryptococcus neoformans* or *Cryptococcus gattii*, with novel cases of *Papiliotrema laurentii* and *Naganishia albida* (Khawcharoenporn et al., 2007; Smith et al., 2017), but it has poor timeliness and low positivity rate for patients receiving antifungal drugs. India ink staining microscopy (India ink) of the CSF is an economical and rapid method, but it has low sensitivity and its performance is affected by the experience of test performers. Cryptococcal capsular polysaccharide antigen (CrAg) test in the CSF is currently the diagnostic assay with the highest sensitivity and specificity (both above 96%) (Boulware et al., 2014), but it cannot determine the presence of infection, detect antigen-deficient strains (Rajasingham et al., 2019), or distinguish specific species. In addition, CrAg antibodies in use are mostly produced by stimulation with *C. neoformans*, which may show low affinity to non-*C. neoformans* strains like *C. gattii* and *P. laurentii*, resulting in decreased sensitivity (99% to 25%) (McMullan et al., 2013; Ragupathi and Reyna, 2015; Smith et al., 2017). Therefore, the method for CM diagnosis is still unsatisfactory. In China, about 40%–66.9% of CM patients are sporadic, HIV-negative, and have no apparent immune deficiency (Lui et al., 2006; Zhu et al., 2010; Liu et al., 2020; Zhang et al., 2020), where it requires more clinical predictions to assign diagnostic tests.

Metagenomic next-generation sequencing (mNGS, also known as high-throughput sequencing) is a genomics-based microbial detection technology developed in recent years (Wilson et al., 2018; Wang et al., 2019; Miller et al., 2019). From 2014 onward, it has been moving gradually from the laboratory toward clinical diagnostic applications, with successful detection of various types of microorganisms such as viruses, bacteria, mycobacteria, fungi, and parasites in clinical samples, showing powerful pathogen detection capabilities. Through alignment to the species-specific sequence of genomes, mNGS can distinguish *C. neoformans* from *C. gattii* and identify coinfections (Xing et al., 2019), which has advantages in identifying strains directly from clinical specimens. Recent studies and case reports with small sample sizes have demonstrated the capability of mNGS to identify fungi like *Cryptococcus* from CSF samples (Wilson et al., 2018; Ramachandran et al., 2019; Wang et al., 2019; Wilson et al., 2019; Xing et al., 2019; Ji et al., 2020; Xing et al., 2020), with preliminarily assessed lower limit of detection (LOD) for *C. neoformans* at about 0.2 CFU/ml (Miller et al., 2019). However, there is yet no systematic evaluation of its performance for the diagnosis of CM, and the clinical significance of mNGS remains unclear.

This study retrospectively recruited HIV-negative patients suspected with acute or subacute central nervous system (CNS) infections whose CSF samples were assigned to both mNGS and routine cryptococcal diagnostic tests (India ink, fungal culture, or CrAg tests) simultaneously in a diagnostic cohort study, with the aim to evaluate the diagnostic performance of mNGS.

## Methods

### Study Design and Subjects

This study is approved by the Medical Ethics Committee of the Third Affiliated Hospital of Sun Yat-sen University [approval no (2021). 02-264-01]. The subjects or the guardians of patients with severe cognitive impairment had provided written consent for research and publication.

In this study, data on 207 Chinese Han HIV-negative cases were screened between July 2018 and December 2019 at the Third Affiliated Hospital of Sun Yat-sen University, Guangzhou, China. Among them, 10 cases were excluded due to loss of information or obvious contamination. Finally, we recruited 197 patients. Inclusion criteria are as follows: 1) Suspected with acute or subacute CNS infection (course duration ≤6 months) with at least one or more of the following clinical manifestations: fever (≥38°C), headache, vomiting, convulsions, meningeal irritation, focal neurological deficits, altered consciousness or lethargy; and at least one of the following conditions should be satisfied: A, abnormal CSF: increased white blood cell (WBC) counts (>5 × 10^6^ cells/ml) and/or increased total protein levels (>0.5 g/L) and/or decreased glucose levels; B, brain imaging suggesting pathological infection or inflammatory changes; 2) CSF samples had been tested by mNGS together with at least one routine cryptococcal diagnostic tests (India ink, fungal culture, or CrAg tests); 3) Written consents of lumbar puncture and mNGS were obtained; 4) Age ≥14 years old. Exclusion criteria are as follows: 1) Incomplete data or loss of follow-up (follow-up ≤1 month); 2) Puncture bleeding; 3) Risk of obvious contamination: mNGS detected more than 2 similar microorganisms (Miller et al., 2019).

### mNGS Procedures and Positivity Standard

A sample of about 2 ml CSF was collected and sealed sterilely and then stored below -20°C or shipped on dry ice to perform PACEseq mNGS test immediately (Hugobiotech, Beijing, China), where technicians had no access to patients’ clinical data. Here, 200 μl of CSF specimen was centrifuged at 5,000g at room temperature for 10 min, and DNA was extracted from the supernatant using a TIANamp Micro DNA Kit (DP316, Tiangen Biotech). A “No template” control (NTC) was also included for each run. The sequencing libraries were constructed *via* QIAseq ™ Ultralow Input Library Kit (Illumina) according to manufacturer’s recommendations. The library concentration and quality were checked using Qubit (Thermo Fisher) and agarose gel electrophoresis. The qualified libraries with different tags were pooled together and amplified and then sequenced by Nextseq550 system (Illumina) for 150 cycles with the high-output Reagent Kit (Illumina) to generate raw data with 5–10 million total reads per sample (Ji et al., 2020).

High-quality data were generated after filtering out adapters and low-quality, low-complexity, and short (<50 bp) reads, and then the human sequences were excluded by mapping reads to the human reference genome (hg19) using Burrows–Wheeler alignment (Li and Durbin, 2009). Finally, to get the microbial compositions of the sample, the remaining data were aligned to the microbial genome database built locally, downloaded from the National Center for Biotechnology Information (ftp://ftp.ncbi.nlm.nih.gov/genomes/), containing genomes of tens of thousands of known microorganisms such as bacteria, archaea, mycoplasma, chlamydia, rickettsia, spirochetes, viruses, and fungi. Besides, the sequencing depth, coverage, and species-specific read number (using genus instead if not matched to any specific species) of each microorganism detected were recorded, and the species-specific read number was further normalized to per megabyte of data and defined as reads per million (RPM), referring to the sequence abundance, and the RPM ratio (RPM-r) was calculated (Miller et al., 2019), defined as RPM_sample_/RPM_NTC_ (if RPM_NTC_ = 0, RPM-r = RPM_sample_).

For *Cryptococcus*, the diagnostic criteria for positive results included the following: 1) The coverage of *Cryptococcus* was in the top 10 of the list of eukaryotes; 2) RPM-r ≥5 [considering the low sequence abundance and low risk of contamination (Bittinger et al., 2014; Schlaberg et al., 2017)] or RPM-r ≥1 (if RPM_NTC_ = 0 and *Cryptococcus* not in the local database of common background microorganisms).

### Conventional Diagnostic Tests and Etiological Diagnosis

The CSF samples of the enrolled patients all underwent blinded mNGS and traditional cryptococcal diagnostic tests according to the routine diagnostic procedures, including 1) fungal culture (Culture); 2) India ink staining microscopy (India ink); 3) lateral flow immunoassays of CrAg (CrAg-LFAs) (Dynamiker Biotechnology, Tianjin, China).

Etiological diagnoses of cases relied on conventional tests performed together but not mNGS. Patients with positive *Cryptococcus* results by culture or India ink were confirmed with cryptococcal infections [1]. For patients with positive CrAg but negative culture and India ink *Cryptococcus* results, other pieces of clinical evidence, including clinical symptoms, CSF test result, outcomes of antifungal treatment, and the possibility of other infection should be considered.

### Statistics and Analysis

Baseline data were collected, and the patients were divided into two groups according to etiological diagnosis: cryptococcal CNS infection and non-cryptococcal infection (viruses, bacteria, immune inflammation, tumor, etc.). The clinical characteristics and the detected *Cryptococcus* species and their RPM levels were compared. The categorical variables were described by the number of cases (percentage). The chi-square test (independent data) was used for comparison between groups. The continuous variables were described in median (lower quartile, upper quartile). The Kruskal–Wallis H test was used for comparison between multiple groups, and the Mann–Whitney U test was used for pairwise comparison. A P-value <0.05 was considered statistically significant. Statistical analysis was performed using SPSS 25.0 software.

## Results

### Baseline Data and Etiological Diagnosis

**Figure 1:**
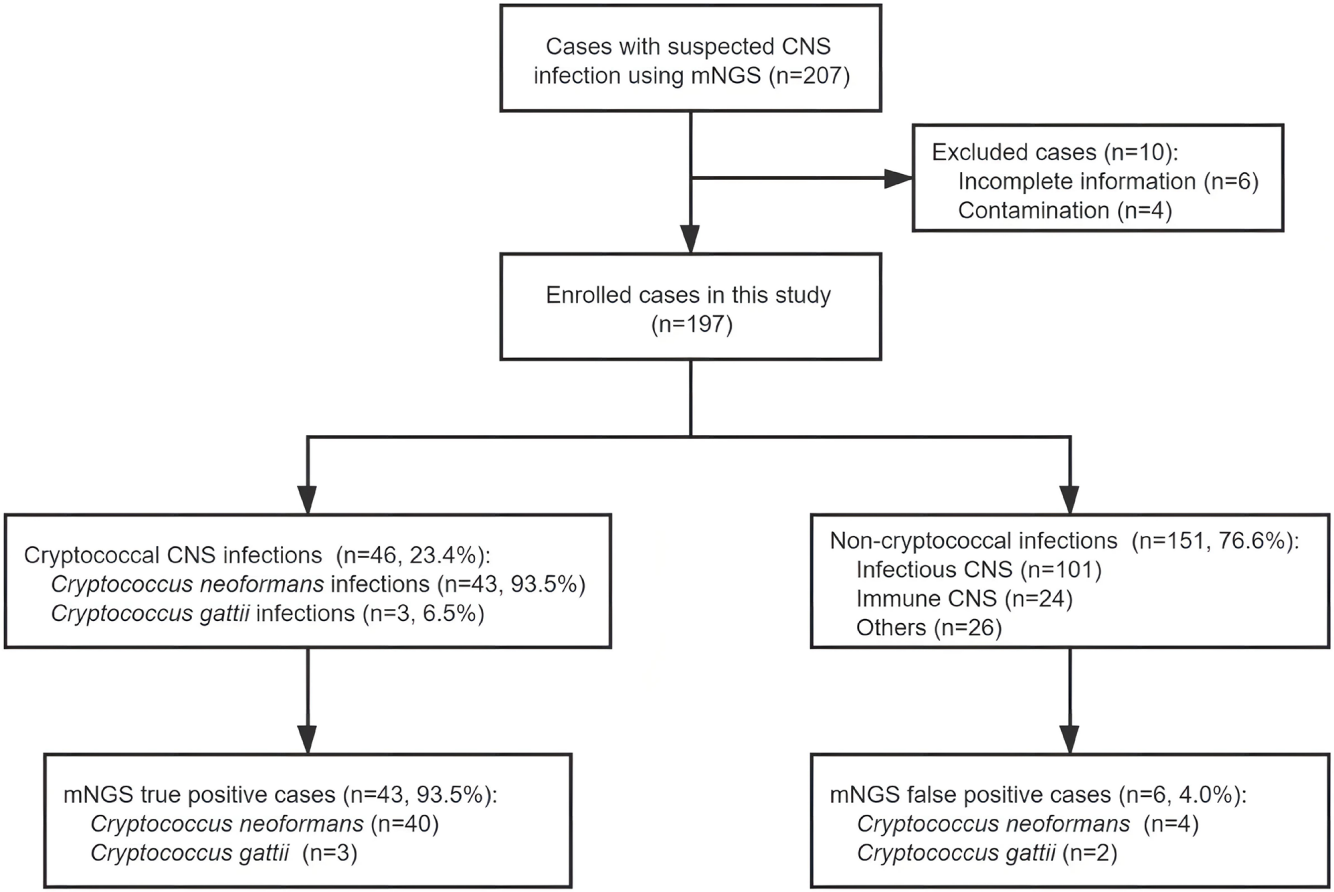
Overview of patient enrollment, metagenomic next-generation sequencing (mNGS) results, and etiological diagnoses. After exclusion of 10 cases, 197 patients with suspected central nervous system (CNS) infection were enrolled in this study. Of these patients, 46 were diagnosed with cryptococcal CNS infection by at least one routine cryptococcal diagnostic test; the other 151 cases were diagnosed with non-cryptococcal infections. The sensitivity and specificity of mNGS in detecting *Cryptococcus* were 93.5% and 96.0%, respectively.

In these 197 cases enrolled in this study (**Figure 1**), 46 (23.4%) cases and 151 (76.6%) cases were etiologically diagnosed as cryptococcal CNS infections and non-cryptococcal infections, respectively. Infections of *C. neoformans* and *C. gattii* accounted for 43 (93.5%) and 3 (6.5%), respectively. The baseline information and clinical data of the enrolled cases are shown in **Table S1**.

### *Cryptococcus* Diagnostic Results

**Figure 2:**
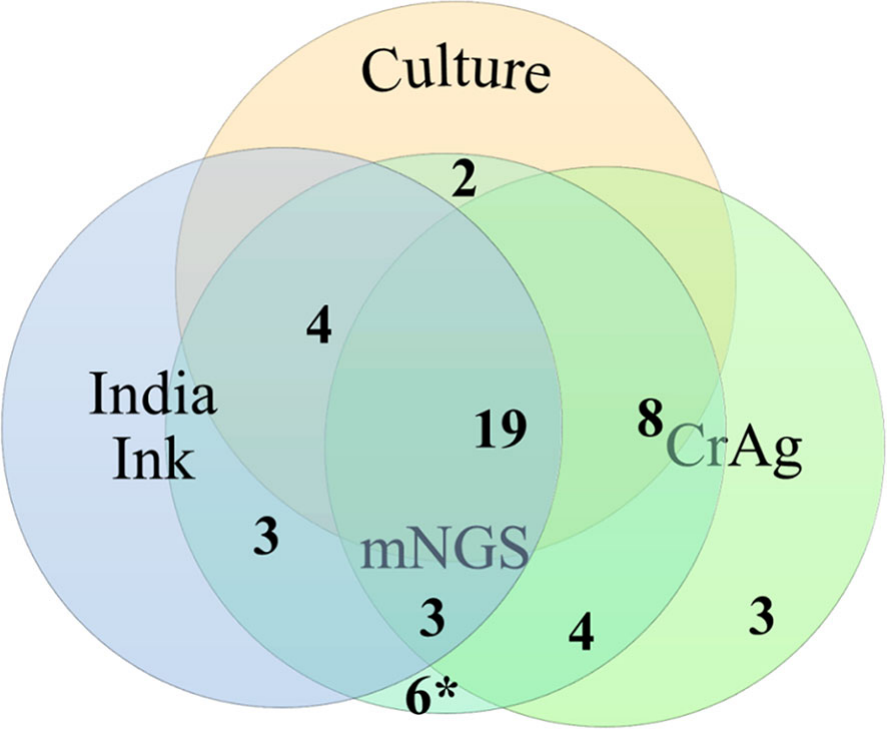
Venn diagram of results of traditional cryptococcal tests and metagenomic next-generation sequencing (mNGS) in 46 patients with cryptococcal central nervous system (CNS) infection. The number of cases detected using culture, India ink, cryptococcal capsular polysaccharide antigen (CrAg), and mNGS was 43, 46, 38, and 46, respectively.*, six mNGS false-positive cases validated by traditional cryptococcal diagnostic tests.

The results of the conventional methods (culture, India ink, and CrAg) and mNGS in the 46 cases diagnosed with CM were shown in **Figure 2**. There were 19 cases diagnosed positive by all of the three traditional methods. Fifteen cases were positive by two traditional methods: 4 (culture and India ink) + 8 (culture and CrAg) + 3 (India ink and CrAg). There were 12 cases with positive results by only one conventional method (2 by culture, 3 by India ink, and 7 by CrAg). It should be noted that the 7 patients diagnosed with CM only by CrAg had corresponding symptoms and abnormal CSF results, which were significantly improved after antifungal treatment, and the possibility of other infectious diseases was ruled out.

### Evaluation of mNGS Diagnostic Performance on Cryptococcal CNS Infections

The sensitivity, specificity, positive predictive value (PPV), negative predictive value (NPV), and concordance rate of mNGS for the diagnosis of CM using CSF were 93.5% [95% confidence interval (CI) at 86.4%~100.0%], 96.0% (95% CI at 92.9%~99.1%), 87.8% (95% CI at 79.0%~96.6%), 98.0% (95% CI at 95.7%~100.0%), and 95.4%, respectively (**Table 1**). These results showed excellent diagnostic power of mNGS, albeit a few false positives.

**Table 1:** The performance of mNGS in the diagnosis of cryptococcal CNS infection.

	Cryptococcal CNS infections (case)		Total (case)	Rate (%)
mNGS	Yes	No		
Cryptococcus (RPM ≥1) +	43	6	49	
Cryptococcus (RPM ≥1) −	3	145	148	
Total	46	151	197	
Sensitivity				93.5 ± 3.6
Specificity				96.0 ± 1.6
Positive predictive value (PPV)				87.8 ± 4.5
Negative predictive value (NPV)				98.0 ± 1.2
Concordance rate				95.4

Cryptococcal CNS infections: at least one routine cryptococcal diagnostic test showing positivity.

CNS, central nervous system; mNGS, metagenomic next-generation sequencing; RPM, reads per million.

The performance of mNGS in diagnosing cryptococcal infections was evaluated by comparing to that of the three conventional methods. The sensitivity, specificity, and concordance rate of the mNGS, India ink, fungal culture, and CrAg methods were shown in **Table 2**. The sensitivity of mNGS was slightly lower than that of CrAg but significantly higher than that of India ink and fungal culture. The specificity of mNGS was not as good as that of the three traditional tests. The concordance rate of mNGS was between that of CrAg and fungal culture.

**Table 2:** Comparison among mNGS and traditional tests for the diagnosis of cryptococcal CNS infection.

	Sensitivity	Specificity	Concordance rate
mNGS	93.5% (43/46) ± 3.6%	96.0% (145/151) ± 1.6%	95.4% (188/197)
India ink	63.0% (29/46) ± 7.1%	100.0% (148/148) ± 0.0%	91.2% (177/194)
Fungal culture	76.7% (33/43) ± 6.4%	100.0% (127/127) ± 0.0%	94.1% (160/170)
CrAg	97.4% (37/38) ± 2.6%	100.0% (44/44) ± 0.0%	98.8% (81/82)

These assays were compared with the composite etiological diagnosis criteria (at least one routine cryptococcal diagnostic test showing positivity).

CNS, central nervous system; CrAg, cryptococcal capsular polysaccharide antigen; mNGS, metagenomic next-generation sequencing.

Culture is considered as the “gold standard” for *Cryptococcus* detection. We further evaluated the mNGS detection results against fungal culture. mNGS reached 100% sensitivity and 100% NPV against culture (**Table 3**), demonstrating its great power in the diagnosis of cryptococcal infections.

**Table 3:** The performance of mNGS against the “gold standard” culture method.

	Culture (case)		Total (case)	Rate (%)
mNGS	Yes	No		
Cryptococcus (RPM ≥1) +	33	13	46	
Cryptococcus (RPM ≥1) −	0	124	124	
Total	33	137	170	
Sensitivity				100.0 ± 0.0
Specificity				90.5 ± 2.5
Positive predictive value (PPV)				71.7 ± 6.6
Negative predictive value (NPV)				100.0 ± 0.0
Concordance rate				92.4

mNGS, metagenomic next-generation sequencing; RPM, reads per million.

## Discussion

To the best of our knowledge, this is the first study to evaluate the diagnostic performance of mNGS for CM in HIV-negative patients. In this study, the sensitivity of mNGS for the diagnosis of cryptococcal infections was slightly lower than that of CrAg but higher than those of India ink and culture, and its specificity was slightly lower than that of the traditional methods, with the concordance rate between that of CrAg and culture. Furthermore, when comparing with the “gold standard” culture method, the sensitivity and NPV of mNGS reached 100.0%, indicating its excellent diagnostic performance. Xing et al. (2019) reported that in 12 cases with India ink- or fungal culture-positive CSF samples, mNGS positivity rate was 75% (9/12), not as good as India ink and fungal culture together (10/12), possibly due to the relatively small sample size.

The false positives of mNGS here were all with very low sequence abundance possibly due to cross-contamination during sampling, as our hospital is a regional CM diagnosis and treatment center in China. It is speculated that false positives would be rare in community hospitals. The false-negative cases were all positive for CrAg assays, and all received antifungal therapies prior to sampling. It could not be excluded that these patients had already cleared the *Cryptococcus* in CSF. After removing the cross-contamination, it was speculated that the sensitivity of mNGS could be comparable to the reported sensitivity (92.9%–96%) of FilmArray (BioMerieux) multiplex PCR for meningitis and encephalitis (Li and Durbin, 2009; Rhein et al., 2016; Liesman et al., 2018), and mNGS could have similar diagnostic value as cryptococcus nucleic acid assays, not just as a screening measure. The sensitivity of mNGS could be improved because the human sequence accounted for more than 95% of the original data, whereas the reads for pathogens were only a small part of it (Simner et al., 2018). To optimize mNGS performance, the next research direction should focus on the efficient removal of human sequences and the enrichment of pathogen sequences (Hasan et al., 2016; Ji et al., 2020). Besides, the collection, transportation, handling of samples, DNA extraction, library construction, procedure standardization, and bioinformatics analysis could all have an impact on the mNGS results (Chiu and Miller, 2019).

mNGS has the following advantages as a diagnosis or screening measure for CM: 1) mNGS has better screening ability and is able to detect mixed infection. mNGS can simultaneously detect a variety of bacteria (including *Mycobacterium tuberculosis*, nontuberculosis mycobacteria) and fungi, without clinical prediction and primer preparation for the presumed pathogens, and it is highly sensitive to *Cryptococcus*. 2) mNGS can assist the diagnosis of atypical cases. In our study, mNGS detected *Cryptococcus* in 7/10 culture-negative samples with India ink or CrAg tests single positive, which offered important support for diagnoses. After prior antifungal treatment, the decline of cryptococcal load and cryptococcal activity or the deficiency of capsular polysaccharide synthesis may lead to the negative results in fungal culture and India ink staining, which may interfere with clinical judgment. In theory, antifungal drugs killed fungi releasing nucleic acid more into the CSF (Miao et al., 2018). Therefore, mNGS may have the advantages in these cases. Secondly, mNGS also has the advantage in detecting DNA from the capsular polysaccharide antigen-deficient strain and non-*C. neoformans* strains. 3) mNGS can directly distinguish *C. neoformans* from *C. gattii*. In China, *C. neoformans* infection is the most prevalent, while *C. gattii* accounts for only 3.4%–7.0% cases (Dou et al., 2015). However, the disease progression and treatment strategy of *C. gattii* infection is significantly different from that of *C. neoformans* infection, such as the higher possibility of neurological complications, the poorer response to multiple antifungal drugs, and the longer period of antifungal treatment (Dou et al., 2015). The traditional strain identification required a positive culture, but fungal culture has a low positivity rate and is very time-consuming, especially after exposure to antifungal drugs. Therefore, it is difficult to guide clinical decision-making. mNGS distinguishes different fungal species by their nucleic acid sequences, simple and fast, providing useful information for diagnosis and treatment. In theory, it could also identify *Papiliotrema laurentii* and *Naganishia albida*; however, these two strains were not detected in this study.

The current cost for mNGS tests is still very high, and the timeliness is not as good as that of India ink staining or CrAg tests. Furthermore, the antifungal drug resistance analysis is not yet available. mNGS could not replace the traditional diagnostic test for CM and is not recommended for routine use. However, it can be used as a good supplementary test because the results have a high degree of credibility and suggest a present infection. In addition, it has advantages in direct strain identification from clinical specimens, which contributes to clinical decisions.

There are several limitations in our study. Firstly, the *Cryptococcus* nucleic acid assays like PCR were not performed to compare the diagnostic ability. Secondly, quite some cases received antifungal treatment before sampling, which might affect the sensitivity to some extent. Furthermore, since the culture for non-*C. neoformans* strains was all negative, we could not assess the accuracy of mNGS to identify *Cryptococcus* strains.

In this study, we evaluated the value of mNGS in the diagnosis of CM in HIV-negative patients. Compared to the conventional methods, mNGS had a higher sensitivity than culture and India ink. The diagnostic ability of mNGS was comparable to that of CrAg method, indicating its excellent diagnostic performance. In addition, mNGS can directly distinguish *C. gattii* from *C. neoforman*s in clinical specimens. Our findings indicated that mNGS using CSF can be considered as a supplementary test to diagnose CM.

## Data Availability Statement

The datasets presented in this study can be found in online repositories. The names of the repository repositories and accession number(s) can be found below: http://ngdc.cncb.ac.cn, PRJCA008890.

## Ethics Statement

The study was conducted according to the principles expressed in the Declaration of Helsinki and approved by the Medical Ethics Committee of the Third Affiliated Hospital of Sun Yat-sen University. All study participants gave written informed consent for research and publication. The patients/participants provided their written informed consent to participate in this study.

## Author Contributions

FP and YJ contributed to the conception and design of this study. ZG, JL, YW, LY, ML, and ZC collected and organized the data. ZG, JL, YW, ZL, HX, YJ, and FP analyzed the data. ZG, JL, YW, LY, ZL, HX, ML, ZC, YJ, and FP drafted the article. All the authors read and approved the final article.

## Funding

This work was supported by the National Science Foundation of Guangdong Province (No. 2015A03013167), the Science and Technology Project of Guangzhou (No. 201510010251), and the Science and Technology Project of Xi’an (No. 21RGSF0013).

## Conflict of Interest

ZL and HX are employed by Hugobiotech Co., Ltd.

The remaining authors declare that the research was conducted in the absence of any commercial or financial relationships that could be construed as a potential conflict of interest.

## Publisher’s Note

All claims expressed in this article are solely those of the authors and do not necessarily represent those of their affiliated organizations, or those of the publisher, the editors and the reviewers. Any product that may be evaluated in this article, or claim that may be made by its manufacturer, is not guaranteed or endorsed by the publisher.
