# Supplementary material for: Performance of Metagenomic Next-Generation Sequencing for the Diagnosis of Cryptococcal Meningitis in HIV-Negative Patients
Source: Front Cell Infect Microbiol. 2022 Apr 21;12:831959. doi: 10.3389/fcimb.2022.831959 (PMC9069553; doi:10.3389/fcimb.2022.831959)
Supplement: Supplementary file 1 [file Table_1.docx]

**Table S1.** Baseline data of patients enrolled

|  | Cryptococcal CNS infections | Non-cryptococcal infections | P value |
| --- | --- | --- | --- |
|  | (n=46) | (n=151) | < 0.05 |
| Male | 31 (67.8%) | 87 (57.8%) | 0.245 |
| Age (year) | 45 (32,60) | 42 (27,54) | 0.231 |
| Immunosuppression or long-term use of corticosteroids | 4 (8.6%) | 10 (6.5%) | 0.083 |
| Course of disease (day) | 32 (22,72) | 22 (13,41) | 0.018 |
| GCS score | 13 (12,15) | 15 (14,15) | 0.038 |
| Anti-fungal treatment before mNGS | 26 (56.5%) | 6 (4.0%) | 0.003 |
| CSF Parameters | | | |
| Open cranial pressure (mmH2O) | 230 (127,314) | 180 (134,240) | 0.149 |
| White blood cell (*10^6^ cells/mL) | 141 (38,283) | 43 (4,143) | 0.001 |
| Proportion of lymphocytes (%) | 82.5 (50.0,90.0) | 88.0 (70.0,94.0) | 0.246 |
| Total protein level (g/L) | 1.02 (0.68,1.76) | 0.56 (0.34,1.13) | 0.004 |
| CSF: Blood glucose ratio | 0.20 (0.06,0.39) | 0.48 (0.35,0.60) | < 0.001 |
| Chloride (mmol/L) | 116.7 (109.6,120.7) | 119.6 (113.2,122.4) | 0.128 |

NOTE: CSF: cerebrospinal fluid. GSC: Glasgow Coma Scale. CNS: central nervous system.
